# High‐Mobility Two‐Dimensional Electron Gas at InGaN/InN Heterointerface Grown by Molecular Beam Epitaxy

**DOI:** 10.1002/advs.201800844

**Published:** 2018-06-27

**Authors:** Tao Wang, Xinqiang Wang, Zhaoying Chen, Xiaoxiao Sun, Ping Wang, Xiantong Zheng, Xin Rong, Liuyun Yang, Weiwei Guo, Ding Wang, Jianpeng Cheng, Xi Lin, Peng Li, Jun Li, Xin He, Qiang Zhang, Mo Li, Jian Zhang, Xuelin Yang, Fujun Xu, Weikun Ge, Xixiang Zhang, Bo Shen

**Affiliations:** ^1^ State Key Laboratory of Artificial Microstructure and Mesoscopic Physics School of Physics Peking University Beijing 100871 P. R. China; ^2^ King Abdullah University of Science and Technology Division of Physical Science and Engineering Thuwal 23955–6900 Kingdom of Saudi Arabia; ^3^ Collaborative Innovation Center of Quantum Matter Beijing 100871 P. R. China; ^4^ Microsystem & Terahertz Research Center China Academy of Engineering Physics (CAEP) Chengdu 610200 P. R. China

**Keywords:** 2D electron gas, InGaN/InN, molecular beam epitaxy

## Abstract

Due to the intrinsic spontaneous and piezoelectric polarization effect, III‐nitride semiconductor heterostructures are promising candidates for generating 2D electron gas (2DEG) system. Among III‐nitrides, InN is predicted to be the best conductive‐channel material because its electrons have the smallest effective mass and it exhibits large band offsets at the heterointerface of GaN/InN or AlN/InN. Until now, that prediction has remained theoretical, due to a giant gap between the optimal growth windows of InN and GaN, and the difficult epitaxial growth of InN in general. The experimental realization of 2DEG at an InGaN/InN heterointerface grown by molecular beam epitaxy is reported here. The directly probed electron mobility and the sheet electron density of the InGaN/InN heterostructure are determined by Hall‐effect measurements at room temperature to be 2.29 × 10^3^ cm^2^ V^−1^ s^−1^ and 2.14 × 10^13^ cm^−2^, respectively, including contribution from the InN bottom layer. The Shubnikov–de Haas results at 3 K confirm that the 2DEG has an electron density of 3.30 × 10^12^ cm^−2^ and a quantum mobility of 1.48 × 10^3^ cm^2^ V^−1^ s^−1^. The experimental observations of 2DEG at the InGaN/InN heterointerface have paved the way for fabricating higher‐speed transistors based on an InN channel.

For decades, 2D electron gas (2DEG) systems have led to important experimental discoveries^1^, ^2^, ^3^, ^4^, ^5^, ^6^, ^7^ and conceptual developments for high‐speed and high‐power electronics.^8^, ^9^ Therefore, searching for materials to host 2DEG has become a key area of research.^10^, ^11^ Researches mainly focus on the heterointerfaces of the heterostructure, e.g., AlGaAs/GaAs,^12^, ^13^ SiO_2_/Si,^14^ LaAlO_3_/SrTiO_3_,^15^ MgZnO/ZnO,^16^ and AlGaN/GaN.^17^, ^18^ The high breakdown voltage, robust thermal stability, high peak electron velocity,^19^ strong polarization effect, and large saturation velocity of III‐nitrides make them excellent materials for hosting high‐mobility 2DEG. GaN‐based 2DEG have already been confirmed at the interfaces of AlGaN/GaN,^2^ InAlN/GaN,^20^ and AlN/GaN,^21^ leading to successful high electron‐mobility transistors (HEMTs) and opening the door to 5G high‐speed wireless communication.^3^, ^22^ Another III‐nitride, InN, has a smaller effective mass and a higher electron‐mobility than GaN, which should make it a better conductive‐channel material for HEMTs.^23^ The theoretically predicted sheet carrier concentration is as high as 7.08 × 10^13^ and 2.12 × 10^14^ cm^−2^ at GaN/InN and AlN/InN interfaces, respectively.^24^ The transconductance and cutoff frequencies of InGaN/InN HEMTs are also predicted to be superior to those of AlGaN/GaN HEMTs.^25^ However, 2DEG in the InGaN/InN heterostructure has remained theoretical, because it is very difficult to epitaxially grow InN and InGaN together in the same structure with high crystallinity and an ultra‐flat surface, due to the giant gap between the optimal growth temperatures of InN and GaN.^26^ The epitaxial growth of InN alone is already challenging.^26^ There are plenty of point defects existing in InN thin film, which can also prevent high quality epitaxy of InN thin film. Precisely detecting the transport properties of 2DEG in an InGaN/InN heterostructure is also experimentally problematic, as two strong, parallel channels of conduction significantly influence the total conductivity: the bottom layer of InN (which is usually degenerated with high electron mobility) and the surface layer of accumulated electrons (which is separated by an InGaN barrier with a high In composition).^27^

Here, we report the experimental observation of 2DEG at the heterointerface of an InGaN/InN heterostructure grown by plasma‐assisted molecular beam epitaxy (PA‐MBE) on an Si substrate. The directly probed electron mobility of the heterostructure, obtained by Hall‐effect measurements at room temperature, was 2.29 × 10^3^ cm^2^ V^−1^ s^−1^, with an electron density as high as 2.14 × 10^13^ cm^−2^. These values include contribution from the bottom InN layer, the InGaN barrier, and the surface layer of accumulated electrons. We measured the multi‐frequency Shubnikov–de Haas (SdH) oscillations of the 2DEG at 3 K, and derived a quantum mobility of 1.48 × 10^3^ cm^2^ V^−1^ s^−1^ with an electron density of 3.30 × 10^12^ cm^−2^. We determined the corresponding quantum scattering time and effective mass of the electrons in the 2DEG at 3 K to be 8.92 × 10^−14^ s and 0.106 *m*_0_, respectively.

The InGaN/InN heterostructures were grown on high resistivity GaN/Si(111) templates by radio‐frequency PA‐MBE; a schematic is shown in **Figure** **1**a. The high resistivity GaN/Si(111) templates were grown by metalorganic vapor‐phase epitaxy. First, the AlN buffer layer was deposited on the Si substrate, followed by an intermediate layer of low Al‐content AlGaN, and then a 3 µm thick layer of GaN was deposited.^28^ Next, the GaN/Si(111) template was transferred to the MBE chamber and a 100 nm thick buffer layer of GaN was grown at 680 °C, followed by a 300 nm thick layer of Mg‐doped InN to suppress the large parallel conductivity of the InGaN/InN heterostructure. Finally, a 70 nm thick layer of undoped InN and a 10 nm thick barrier layer of InGaN were grown at 500 °C with a 96% In composition. The Mg cell temperature was kept at 250 °C during the growth of the Mg‐doped InN to create a p‐type conductive material, which should reduce the contribution of the undoped InN layer to the conductivity of the total heterostructure.^29^ Atomic force microscopy (AFM) measurements revealed an atomically flat surface with step terraces, as shown in Figure 1b. The root mean square roughness was as small as 0.7 nm in a scanned area of 1 × 1 µm^2^. The InGaN/InN heterostructure was also characterized by X‐ray diffraction (XRD), with the fitting curve compared to the measured spectrum (Figure S1, Supporting Information) verifying that the In content was about 96%, in good agreement with our design. Figure 1c shows the XRD reciprocal space map for the (105) reflection of the InGaN/InN heterostructure. Both the InGaN and InN diffraction peaks are located in the same position of reciprocal lattice vector *Q_x_*, indicating that the InGaN layer was coherently grown on the underlying InN layer. This 10 nm thick, coherent epitaxial growth of InGaN on InN was possible due to the small lattice mismatch (0.4%) between InN and high In‐content InGaN. To characterize the heterostructure of InGaN/InN directly, we performed high‐resolution scanning transmission electron microscopy experiments. The image in Figure 1d was obtained on an aberration‐corrected scanning transmission electron microscope (STEM). To further confirm the epitaxial growth and structure, electron energy‐loss spectroscopy (EELS) mapping was performed for Ga in the region marked with a red dashed square in Figure 1d. The thickness of the InGaN barrier was about 10 nm, in good agreement with the thickness designed during MBE.

**Figure 1:**
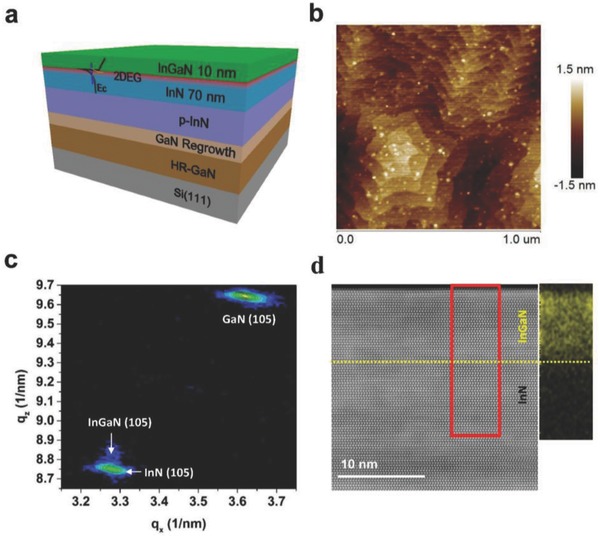
Schematic of the heterostructure and surface morphology. a) Sketch of the InGaN/InN heterostructure with the band profile at the InGaN/InN interface. b) AFM topography (1 × 1 µm^2^) of the InGaN/InN heterostructure. c) Reciprocal space map of the InGaN/InN heterostructure for (105) plane reflection. d) High‐angle annular dark‐field scanning transmission electron microscopy (HAADF‐STEM) image of the InGaN/InN heterostructure; magnified and spatially resolved EELS mapping of Ga shows that the InGaN layer is 10 nm thick.

In order to obtain information about the 2DEG in the heterostructure, Hall‐effect measurements were performed at different temperatures with van der Pauw geometry and the four‐probe AC technique on a physical property measurement system (PPMS). **Figure** **2** shows the temperature dependence of the electron sheet concentration and mobility across a temperature range of 3–300 K. The electron mobility was as high as 2.29 × 10^3^ cm^2^ V^−1^ s^−1^ at 300 K, and reached a maximum value of 4.80 × 10^3^ cm^2^ V^−1^ s^−1^ at 3 K. The weak temperature dependence of the electron mobility in the temperature range of 3–100 K strongly indicates that the electric conductivity was dominated by 2DEG at low temperatures. The low temperature mobility of 2DEG in the InGaN/InN heterostructure is smaller than AlGaN/GaN heterostructure because InGaN/InN heterostructure with ultra‐thin InN thin film is accompanied by high dislocation densities. We have roughly estimated dislocation densities of InN in the InGaN/InN heterostructure from the full width at half maximum (FWHM) of the rocking curves on InN (002) and (102) planes. The density of screw‐ and edge‐type threading dislocation of InN is 5.88 × 10^8^ cm^−2^ and 7.23 × 10^10^ cm^−2^, respectively (see Figure S1b, Supporting Information). Those high density threading dislocations definitely limit the mobility of the 2DEG in the InGaN/InN heterostructure as well since the threading dislocations extend to the barrier with growth. Therefore, the mobility of 2DEG in the InGaN/InN heterostructure is strongly affected by the high dislocation densities. In addition, the residual electron concentration for the InN layer is about 3.6 × 10^18^ cm^−3^. It indicates that a large density of ionized impurities exists in the sample, which greatly limits the mobility of the 2DEG as well. The mobility of heterostructure can definitely be enhanced if we are able to reduce the ionized impurity and ionized dislocation density. To exclude the possibility that the high mobility of the InN layer was responsible, we measured the transport properties of bare InN film (without the InGaN barrier). The electron mobility of InN decreases rapidly with decreasing temperatures when *T* < 115 K (see Figure S2, Supporting Information), which is a typical behavior of InN thin film materials but not 2DEG, and in contrast to the behavior shown in Figure 2. Therefore, at low temperatures, the electric conduction of the heterostructure was indeed dominated by 2DEG. A weak temperature dependence was also obtained for the sheet electron concentration, which gradually decreased from ≈2.14 × 10^13^ cm^−2^ at room temperature to 1.64 × 10^13^ cm^−2^ at 3 K. This suggests that the defect levels responsible for generating the carriers become degenerated at low temperatures.^30^

**Figure 2:**
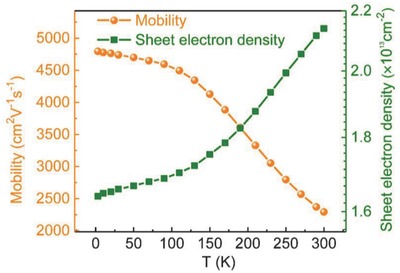
Temperature‐dependent electronic properties of the InGaN/InN heterostructure. The low‐field Hall mobility and carrier density as functions of temperature with magnetic fields ranging from −0.5 to 0.5 T; the mobility reaches 4.80 × 10^3^ cm^2^ V^−1^ s^−1^ at 3 K.

To fully characterize the electric transport properties of 2DEG in the heterostructure, we performed magnetoresistance measurements using a standard lock‐in technique, with an AC excitation of 100 µA and magnetic fields applied perpendicular to the surface. Curves representing the resistance versus the magnetic field at different temperatures are shown in **Figure** **3**a. Overall, the magnetoresistance increased with the magnetic field, and did not show trends of saturation up to 14 T, which might be ascribed to the parallel conductive channels in the heterostructure.^31^ The magnetoresistance dependence of magnetic field *R_xx_*(*B*) curves exhibit some oscillations at low temperatures. As temperature increases, the oscillations become weaker until they vanish at 50 K. By fitting the experimental data, we found that the *R_xx_*(*B*) curves reflect a positive magnetoresistance background at low temperatures, which can be described by a *B* ^3^ polynomial function.^32^ We found that the *B* ^3^ polynomial function worked best to subtract the background. The magnetoresistance SdH oscillation value of Δ*R_xx_* = *R_xx_* (*B*) – *αB* ^3^ is shown in Figure 3b, where *R_xx_*(*B*) represents the data presented in Figure 3a and α > 0 is a fitting parameter.

**Figure 3:**
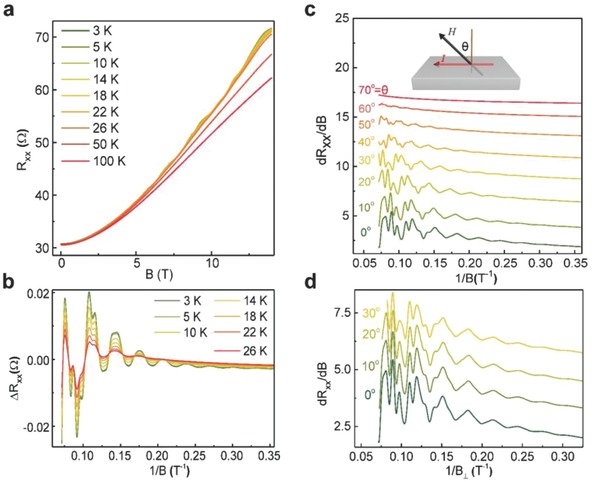
Temperature and angle‐dependent SdH oscillation. a) Magnetoresistance (*R_xx_*) as a function of the magnetic fields at different temperatures. b) Oscillation amplitude as a function of the reciprocal magnetic fields at different temperatures. c,d) Amplitude of the SdH oscillations of the first derivative, *dR_xx_/dB*, at different angles θ versus the reciprocal magnetic field c) and the reciprocal perpendicular magnetic field d), respectively. The SdH oscillations depend mainly on the reciprocal perpendicular magnetic fields component, particularly for θ = 0°–30°, which suggests a 2D nature of conduction at the interface of the InGaN/InN heterostructure. Inset: Measurement configuration.

Some of the SdH oscillations were likely caused by 2DEG. To confirm their origin, we studied the behavior of the SdH oscillations following the application of magnetic fields at different orientations. The derivative of the magnetoresistance (*dR_xx_/dB*) is shown in Figure 3c,d as a function of 1/*B* and 1/*B*_⊥_, respectively (*B*_⊥_ = *B*cosθ, where the angle θ = 0° corresponds to a magnetic field applied perpendicular to the film plane). As we increased the angle θ, the peak positions shifted to lower values of 1/*B*, i.e., larger values of *B* (Figure 3c). When we increased the angle θ to 70°, we observed no oscillation with fields up to *B* = 14 T. However, when *dR_xx_/dB* was plotted as a function of 1/*B*_⊥_ (Figure 3d), all of the well‐defined peaks appeared at the same position in curves of θ ≤ 30°, indicating that the oscillations depend only on the perpendicular application of the magnetic fields. The results in Figure 3d unambiguously demonstrate that the electron gas in our sample is 2D. Evidence of the 2D nature of the SdH oscillations is also revealed by the change in magnetoresistance at different angles (see Figure S3, Supporting Information).

We performed more detailed analysis on the data to more deeply understand the properties of the 2DEG in the InGaN/InN heterostructures. We carried out a fast Fourier transform (FFT) analysis of the SdH oscillation data from Figure 3b, shown in **Figure** **4**a. We observed four peaks with frequencies of 30.20, 68.08, 97.68, and 147.74 T. The relationship between carrier density and oscillation frequency can be described by the formula: *n_i_* = *eB_Fi_*/πℏ(*B_F_* is SdH oscillation frequency). The carrier densities corresponding to those four peaks are *n_α_* = 1.47 × 10^12^ cm^−2^, *n_β_* = 3.30 × 10^12^ cm^−2^, *n_γ_* = 4.74 × 10^12^ cm^−2^, and *n_δ_* = 7.17 × 10^12^ cm^−2^, respectively, which we calculated using the free electron approximation model, assuming that the electrons were spin‐degenerated.^33^ We ascribed the discrepancy between the carrier densities deduced from the Hall‐effect measurements (2.09 × 10^13^ cm^−2^) and the SdH oscillations (1.19 × 10^13^ cm^−2^) to the parallel conductive channels in the InGaN/InN heterostructure. Also, the sheet carrier density deduced from the Hall effect is based on averaged values, whereas the sheet carrier density obtained from the SdH oscillation is based on electron moves on the surface of the Fermi sphere. We also calculated the carrier effective mass, m*, and the quantum scattering time, *τ_q_*, using the temperature dependence of the SdH oscillation amplitudes, Δ*R*,^34^, ^35^ with the formula ∆*R_xx_*∝e^−α^ *T*_*D*_ *αT*/sin h(*αT*), where α = *2π^2^k*_B_ m*/ℏeB, *k*_B_ is the Boltzmann constant, m* is the effective mass of the carriers, and *αT/*sinh*(αT)* is the thermal damping. The Dingle temperature factor, *T_D_ = ℏ/2πk*_B_ *τ_q_*, corresponds to the level of disorder and can be used to obtain the quantum scattering time *τ_q_* through the following formula

$$-A\cdot T_{D}/B=\ln\left[\frac{\Delta R\cdot \sin\;h(A\cdot T/B)}{4R_{0}\cdot A\cdot T/B}\right]\propto \ln\left[\frac{\Delta R\cdot \sin\;h(A\cdot T/B)}{A\cdot T/B}\right].$$

**Figure 4:**
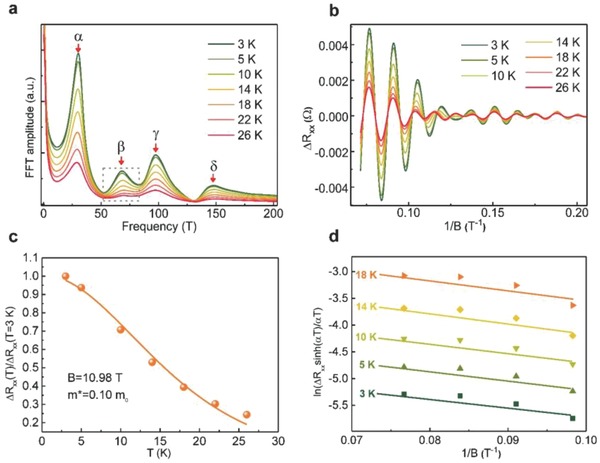
β peak IFFT process and carrier effective mass. a) FFT spectra of the SdH oscillations from Figure 3b. Arrows indicate the different peaks, α, β, γ, and δ. b) IFFT curves for the β peak from the FFT spectra. c) Temperature dependence of the normalized oscillation amplitude at *B* = 10.99 T, giving an electron effective mass of 0.10 *m*_0_. d) Dingle plots of ln[∆*R_xx_*sinh(*αT*)/*αT*] versus *B* ^−1^ at different temperatures (θ = 0°).

However, as shown in Figure 3b, all of the main peaks were amplified by the overlapping peaks of different frequencies, which prevented us from obtaining the amplitudes of the original SdH oscillations. Therefore, we analyzed the temperature‐dependent SdH oscillations instead, shown in Figure 4b, which were constructed from the inverse fast Fourier transform (IFFT) of the β peak (Figure 4a).^36^ The deviation of the magneto‐transport traces between 0.1 and 0.15 1/*T* mainly results from two aspects: 1) the β peak shape slightly changes with increasing temperature and, 2) the β peak is partially overlapped with the side peaks (γ peak and α peak) rather than being a completely independent peak. The related works reported by other groups also show similar behavior over the IFFT.^5^ The most essential issue is that the magneto‐transport traces for different temperatures can match well with the oscillation peak. Thus, we obtained *m_β_* = (0.106 ± 0.003) *m*_0_ for the β peak from the temperature‐dependent amplitudes of the SdH oscillations (Figure 4c).

The resulting Dingle plots for the β peak at different temperatures are shown in Figure 4d. The quantum scattering time, *τ_β_* = 8.92 × 10^−14^ s, was deduced from the slope of the linear dependence of ln[∆*R_xx_*sinh(*αT*)/*αT*] on 1*/B*. The quantum mobility, *µ_β_* = eτ/m* = 1.48 × 10^3^ cm^2^ V^−1^ s^−1^, was obtained using *τ_β_* = 8.92 × 10^−14^ s. The classical scattering time obtained from the Hall‐effect measurement was τ_0_ = 1.91 × 10^−13^ s. Thus, the ratio τ_0_/τ_β_ ≈ 2 suggests that the short‐range alloy‐disorder scattering and the rough‐interface scattering are the dominant factors limiting electron mobility at low temperatures.^37^ The quantum mobility and the Hall mobility are different in the InGaN/InN heterostructure, which may be caused by the different scattering times, i.e., the transport scattering time and the total scattering time, respectively. Therefore, in order to achieve high‐mobility 2DEG in the InGaN/InN heterostructure, it is important to control the interface roughness and alloy‐disorder scattering. As a ternary alloy, InGaN layer exhibits alloy‐disorder scattering. Dislocation scattering is also an important mobility‐limiting scattering mechanism in 2DEGs when the dislocation density is high.^38^ Therefore, low dislocation density is vital to high‐performance InN thin film and InN‐based 2DEG. The effective masses and quantum scattering lifetimes of the other three peaks were also obtained using the same methodology: *m_α_* = (0.079 ± 0.003) *m*_0_, *m_γ_* = (0.089 ± 0.002) *m*_0_, and *m_δ_* = (0.102 ± 0.001) *m*_0_; and *τ_α_* = 5.14 × 10^−14^ s, *τ_γ_* = 4.63 × 10^−14^ s, and *τ_δ_* = 2.78 × 10^−14^ s, respectively. The detailed calculations are shown for each peak in Figures S4, S5, and S6 (Supporting Information), respectively.

To reveal the mechanism behind the quantum oscillations in the InGaN/InN heterostructure, we determined the origin of the four oscillation frequencies. The effective mass of peak α, *m_α_* = 0.079 *m*_0_, is similar to that of InN, *m*_InN_ = 0.070 *m*_0_.^39^ Therefore, peak α most likely originated from the InN layer. For confirmation, we measured the transport of a bare InN film without the InGaN barrier. After removing the positive magnetoresistance background described above from the experimental data, we observed a clear SdH oscillation at 2 K. An FFT analysis of the SdH oscillation revealed that this SdH oscillation corresponded to a frequency of 34.30 T (see Figure S7, Supporting Information), which is almost the same as the frequency of peak α observed in the InGaN/InN heterostructure. Therefore, we ascribe the origin of peak α in the InGaN/InN heterostructure to the underlying InN layer. The electron effective mass *m_β_* of peak β was heavier than *m_α_*; hence, it probably originated from 2DEG at the InGaN/InN interface. The heavier *m_β_* compared to *m_α_* could be ascribed to both the nonparabolicity of the conduction band in the triangular quantum well and to the penetration of the electron wave function into the barrier.^40^ Peak γ was the sum of peaks α and β (*B_Fγ_ = B_Fα_ + B_Fβ_*), indicating the existence of the magnetic breakdown effect.^41^ Peak δ, which had the highest frequency, most likely arose from the top InGaN layer, given its high electron sheet density of 7.17 × 10^12^ cm^−2^, which included contribution from the surface layer of accumulated electrons.^27^ Four peaks observed at FFT spectra of the SdH oscillations actually originated from InN bulk, 2DEG, 2DEG+InN bulk, and InGaN barrier, respectively.

We also simulated the subband occupation of the 2DEG in the InGaN/InN heterostructure. We carried out the simulations based on the Schrödinger and Poisson equations and advanced physical models using Crosslight Software, Inc.'s APSYS program. The numerical results (Figure S8, Supporting Information) indicate that only one subband was occupied below the Fermi energy level and that the gap between the Fermi energy level and the subband was 68.84 meV, which is close to the aforementioned experimental fitting result of ≈74.99 meV. The discrepancy between these two values might be due to the unexpected InGaN accumulation of electrons on the surface that was not taken into account during the simulation. In addition, as was reported for InN,^42^ the energy level differences between the Fermi level and the corresponding subbands induced by electron accumulation on the surface were 511 and 797 meV, respectively, which are much larger than those that originate from 2DEG. Therefore, we exclude the possibility that the electron accumulation on the heterostructure surface induces a subband occupation. This result further confirms that the β peak is originated from 2DEG at the InGaN/InN heterointerface.

In conclusion, we clearly observed quantum magnetoresistance oscillations at low temperatures in the InGaN/InN heterostructures that stemmed from 2DEG formed at the InGaN/InN heterointerface. We found that at 3 K, the quantum mobility and electron density of the 2DEG were 1.48 × 10^3^ cm^2^ V^−1^ s^−1^ and 3.30 × 10^12^ cm^−2^, respectively. This experimental observation of 2DEG at the InGaN/InN heterointerface invites further investigation of III‐nitride semiconductor materials for higher‐speed transistors.

## Experimental Section

*Sample Growth and Transport Measurements*: All heterostructures were grown by plasma‐assisted molecular beam epitaxy (PA‐MBE, SVTA). Gallium (Ga), and indium (In) fluxes were evaporated from pure elemental sources. Reactive atomic nitrogen (N) was generated by radio frequency plasmas to dissociate the N atoms with 1.20 sccm. The chamber pressure was kept at 7.00 × 10^−6^ Torr during growth. The InN and InGaN layers were grown at a substrate temperature of 500 °C, during which the growth process was monitored in situ by reflection high‐energy electron diffraction. The Ga content was controlled by the Ga/In flux ratio. The transport properties were measured using the four‐probe van der Pauw method, with copper (Cu) wires as the electrodes and In as the ohmic contact. The Hall effect was measured in the magnetic field range of −0.5 to 0.5 T. Electrical measurements were performed using a PPMS DynaCool system (temperatures from 50 mK to 400 K, and magnetic fields up to 14 T) with a standard lock‐in technique.

*Heterostructure Characterization Analysis*: The surface of the InGaN/InN heterostructures was characterized with a Bruker Dimension FastScan AFM in the tapping mode. Aberration‐corrected STEM measurements were performed with an FEI Titan 80–300 Probe TEM. The high‐angle annular dark‐field imaging (HAADF) images were acquired at 200 kV. A cross‐sectional sample was cut by an FEI Helios DualBeam FIB system. STEM electron energy‐loss spectroscopy (STEM‐EELS) was used to investigate the spatial distribution of the Ga elements on the InGaN surface. In order to improve the signal‐to‐noise ratio of the EELS spectra, principal components analysis was used to reduce the noise.

## Conflict of Interest

The authors declare no conflict of interest.

## Supporting information

Supplementary
