# Supplementary material for: High‐Mobility Two‐Dimensional Electron Gas at InGaN/InN Heterointerface Grown by Molecular Beam Epitaxy
Source: Adv Sci (Weinh). 2018 Jun 27;5(9):1800844. doi: 10.1002/advs.201800844 (PMC6145405; doi:10.1002/advs.201800844)
Supplement: Supplementary file 1 — Supplementary [file ADVS-5-1800844-s001.pdf]

Copyright WILEY-VCH Verlag GmbH & Co. KGaA, 69469 Weinheim, Germany, 2018.

ADVANCED SCIENCE
Open Access

Supporting Information

for *Adv. Sci.,* DOI: 10.1002/advs.201800844

High-Mobility Two-Dimensional Electron Gas at InGaN/InN Heterointerface Grown by Molecular Beam Epitaxy

Tao Wang, Xinqiang Wang, Zhaoying Chen, Xiaoxiao Sun, Ping Wang, Xiantong Zheng, Xin Rong, Liuyun Yang, Weiwei Guo, Ding Wang, Jianpeng Cheng, Xi Lin, Peng Li, Jun Li, Xin He, Qiang Zhang, Mo Li, Jian Zhang, Xuelin Yang, Fujun Xu, Weikun Ge, Xixiang Zhang,* and Bo Shen**

Copyright WILEY-VCH Verlag GmbH & Co. KGaA, 69469 Weinheim, Germany, 2017.

Supporting Information

Realization of High-Mobility Two-Dimensional Electron Gas at InGaN/InN Heterointerface

Tao Wang, Xinqiang Wang, Zhaoying Chen, Xiaoxiao Sun, Ping Wang, Xiantong Zheng, Xin Rong, Liuyun Yang, Weiwei Guo, Ding Wang, Jianpeng Cheng, Xi Lin, Peng Li, Jun Li, Xin He, Qiang Zhang, Mo Li, Jian Zhang, Xuelin Yang, Fujun Xu, Weikun Ge, Xixiang Zhang*, and Bo Shen**

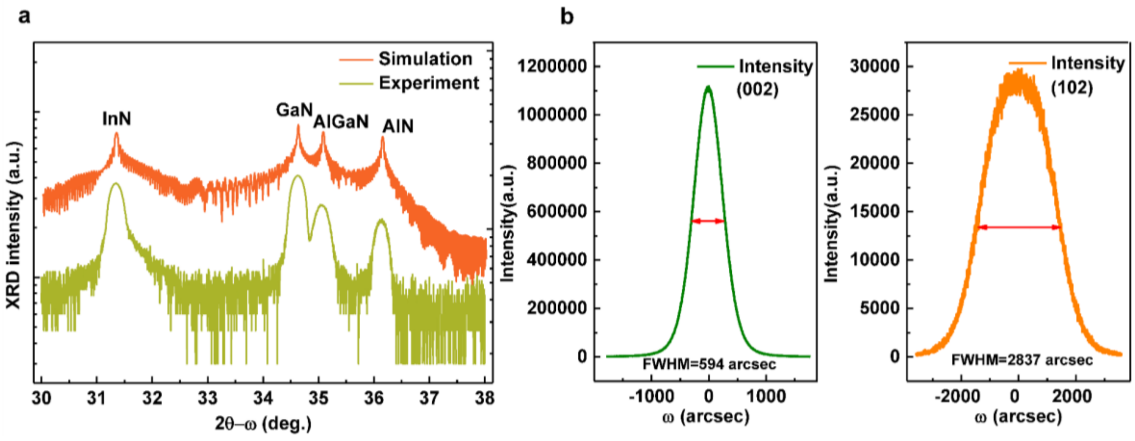

Figure S1. X-ray diffraction spectra of the InGaN/InN heterostructure grown on Si substrate. (a) The XRD *2θ-ω* spectrum (yellow-green) measured across the (0002) reflection plane of the InGaN/InN heterostructure. The simulated spectrum (red) clearly agrees well with the experimental data. **(b)** The XRD rocking curves of InN channel layer (002) plane (green) and (102) planes (pink) in the InGaN/InN heterostructure.

High-resolution X-ray diffraction (HRXRD) *2θ-ω* scans of the symmetric (0002) plane were performed on the sample using a Bruker D8 Advanced HRXRD system. The asymmetric diffraction peak in InN was induced by the thin InGaN barrier with a low Ga content. The simulation results indicate that the InGaN barrier is 10 nm thick with a Ga content of 4%. The FWHM of the rocking curves on InN (002) and (102) planes and the estimated dislocation densities of InN in the InGaN/InN heterostructure. The screw dislocation of InN is 5.88×10^{8} cm^{-2}, and edge dislocation densities in InN is 7.23×10^{10} cm^{-2}.

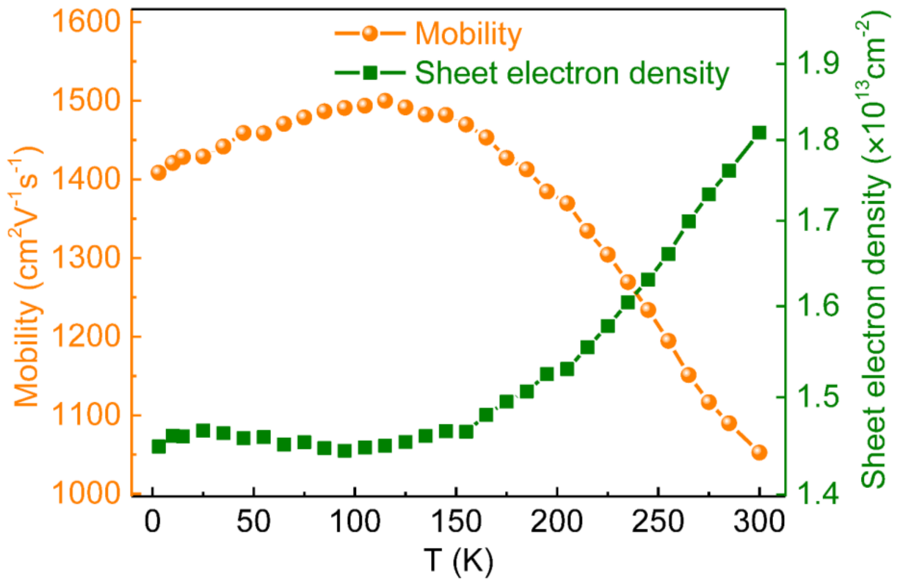

Figure S2. Temperature-dependent Hall mobility (spheres) and sheet electron concentration (squares) of bulk InN, measured under magnetic fields ranging from -0.5 to 0.5 T.

To exclude the possibility that the properties of the InGaN/InN heterostructure presented in Figure. 2 (main text) were caused by the high mobility of the InN bottom layer, we analyzed the temperature dependence of the Hall mobility and sheet electron concentration of bare InN film in a physical properties measurement system (PPMS). As shown in Figure. S2, the electron mobility of InN rapidly decreased with decreasing temperatures when $T<115$ K because the ionized impurity scattering dominates in the low temperature range from 2 K to 115 K. However, the lattice scattering becomes dominant when the temperature is higher than 115 K, which explains why the electron mobility also decreased with increasing temperature.[1]

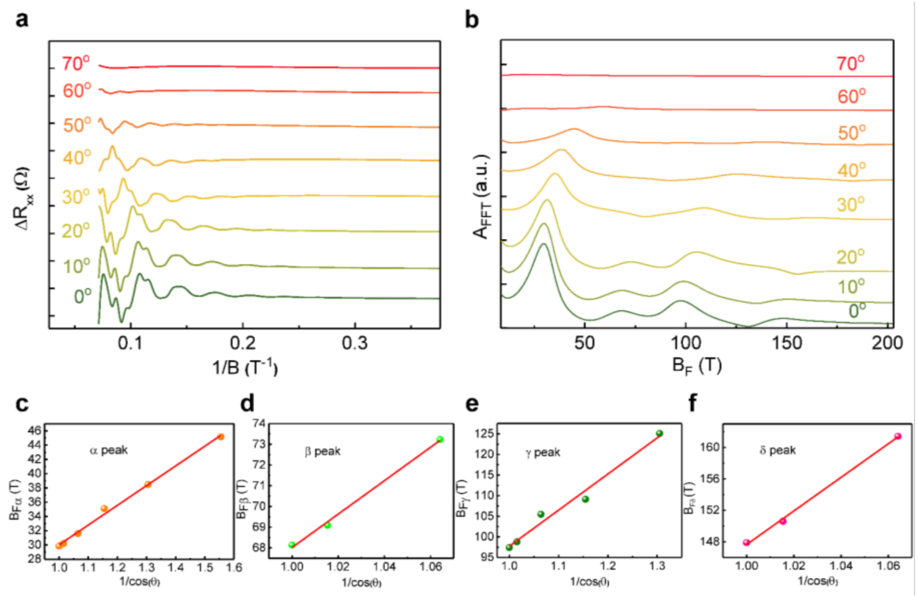

Figure S3. Angular dependence of the SdH oscillations obtained from an InGaN/InN heterostructure. (a) The magnetoresistance SdH oscillation curves ($\Delta R_{xx} = R_{xx}(B) - \alpha B^3$) as a function of $1/B$ for different angles θ obtained at 3 K. **(b)** The fast Fourier transform (FFT) of the SdH oscillations shown in **(a)**. The peaks correspond to different Fermi surfaces. The SdH oscillation frequencies B_F (i.e., $B_{F\alpha}$ **(c)**, $B_{F\beta}$ **(d)**, $B_{F\gamma}$ **(e)**, and $B_{F\delta}$ **(f)**) were extracted from **(b)** and are plotted as a function of $1/\cos\theta$.

In the main text, we studied the behavior of the SdH oscillations when a magnetic field was applied at different orientations, as shown in Figure. 3c-d. To further probe the two-dimensional character of the conduction, we performed magnetoresistance experiments with the magnetic field applied at different angles. Figure. S3a shows the amplitude of the SdH oscillations ($\Delta R_{xx} = R_{xx}(B) - \alpha B^3$) versus the reciprocal magnetic field (1/B) obtained at 3 K and at different angles. The peak positions shifted to lower values of 1/B as the angle θ was increased. When the angle θ was increased to 70°, no oscillation was observed. Figure. S3b shows the fast Fourier transform (FFT) data of the SdH oscillations from Figure. S3a. The peaks were obviously reduced by increasing the angle θ and completely vanished at θ =70°. The SdH oscillation frequencies B_F ($B_{F\alpha}$, $B_{F\beta}$, $B_{F\gamma}$, and $B_{F\delta}$), extracted from Figure. S3a, were plotted as functions of $1/\cos(\theta)$ in Figure. S3c-f. The linear dependence of the data strongly indicates the two-dimensional nature of conduction in the

InGaN/InN heterostructures with multiple conduction channels. Unlike peak α, peak β was not observed when the angle θ increased to 40°, indicating that the two-dimensional confinement of peak β was strong compared to that of peak α.

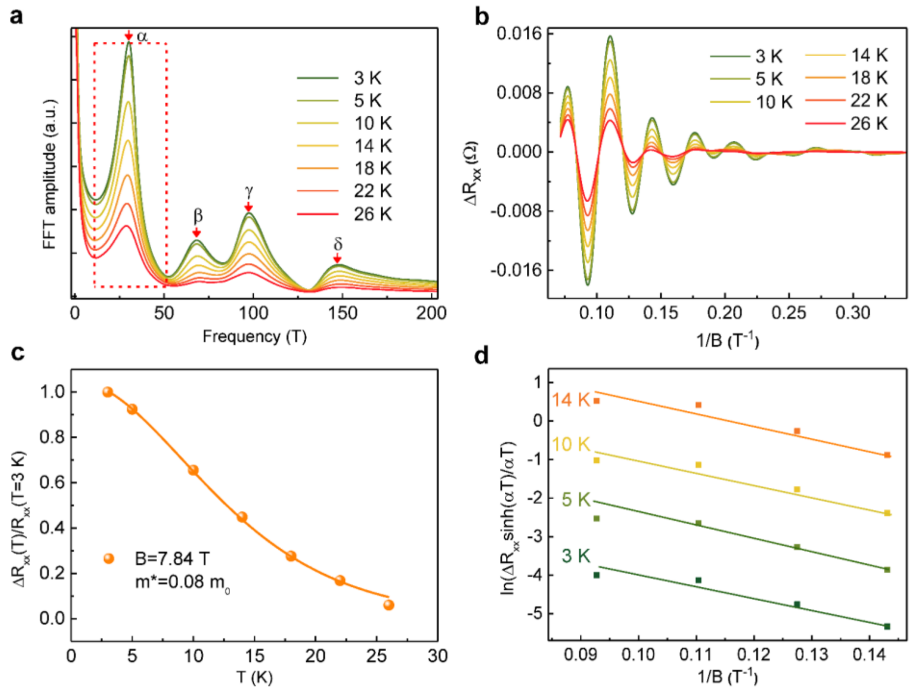

Figure S4. IFFT and carrier effective mass corresponding to peak *α*. (a) FFT spectra of the SdH oscillations at various temperatures, which are also shown in Figure. 3b (main text). The arrows indicate the different peaks α, β, γ, and δ. **(b)** IFFT curves for peak α at different temperatures. **(c)** Temperature dependence of the normalized oscillation amplitude at frequency B = 7.84 T, giving an electron effective mass of 0.08 m_0. **(d)** Dingle plots of $\ln[\Delta R_{xx}\sinh(\alpha T)/\alpha T]$ vs. $1/B$ at different temperatures ($\theta = 0^o$).

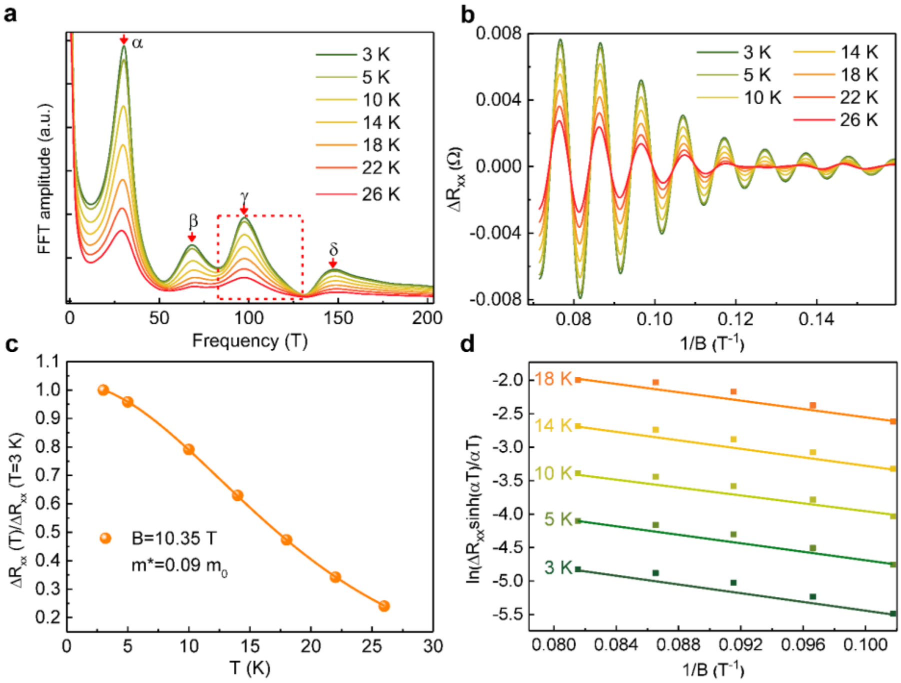

Figure S5. IFFT and carrier effective mass corresponding to peak γ. (a) FFT spectra of the SdH oscillations at various temperatures, which are also shown in Figure. 3b (main text). The arrows indicate the different peaks α, β, γ, and δ. **(b)** IFFT curves for peak γ at different temperatures. **(c)** Temperature dependence of the normalized oscillation amplitude at frequency B = 10.35 T, giving an electron effective mass of 0.09 m_0. **(d)** Dingle plots of ln[ΔR_{xx}sinh(αT)/αT] vs. 1/B at different temperatures (θ = 0°).

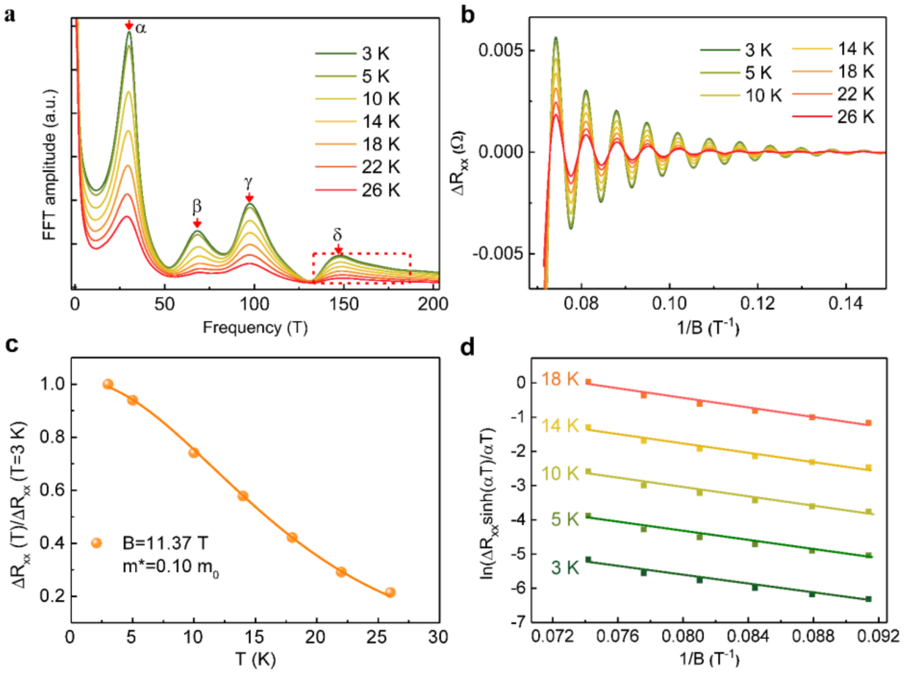

Figure S6. IFFT and carrier effective mass corresponding to peak *δ*. (a) FFT spectra of the SdH oscillations at various temperatures, which are also shown in Figure. 3b (main text). The arrows indicate the different peaks *α*, *β*, *γ*, and *δ*. **(b)** IFFT curves for peak *δ* at different temperatures. **(c)** Temperature dependence of the normalized oscillation amplitude at frequency $B = 11.37$ T, giving an electron effective mass of 0.10 m_0. **(d)** Dingle plots of $\ln[\Delta R_{xx}\sinh(\alpha T)/\alpha T]$ vs. $1/B$ at different temperatures ($\theta = 0^o$).

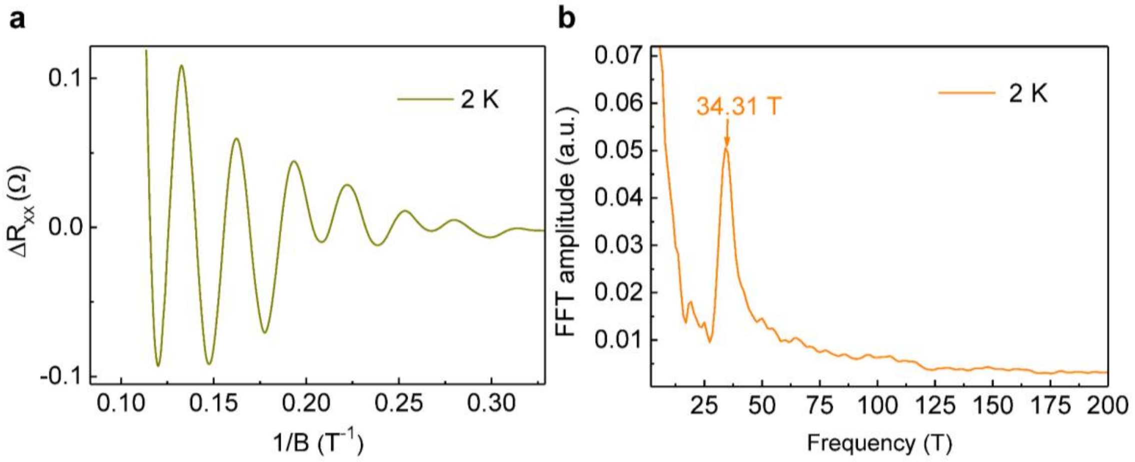

Figure S7. SdH oscillations of an InN thin film. (a) Magnetoresistance SdH oscillation curves ($\Delta R_{xx} = R_{xx}(B) - \alpha B^3$) as a function of $1/B$ for InN measured at 2 K. **(b)** FFT of the data from **(a)**. The obvious peak located at 34.31 T corresponds to peak *α* in the InGaN/InN FFT spectrum.

To confirm that peak *α* originated from the InN layer, we measured the transport properties of bare InN film. After removing the positive magnetoresistance background using a B^3 polynomial function,[2] we observed an obvious SdH oscillation at 2 K. The FFT analysis revealed that this SdH oscillation in InN corresponded to a frequency of 34.30 T, as shown in Figure. S7. This is almost the same as the lowest frequency of peak *α* observed in the InGaN/InN heterostructure, confirming that peak *α* originated from the underlying InN layer.

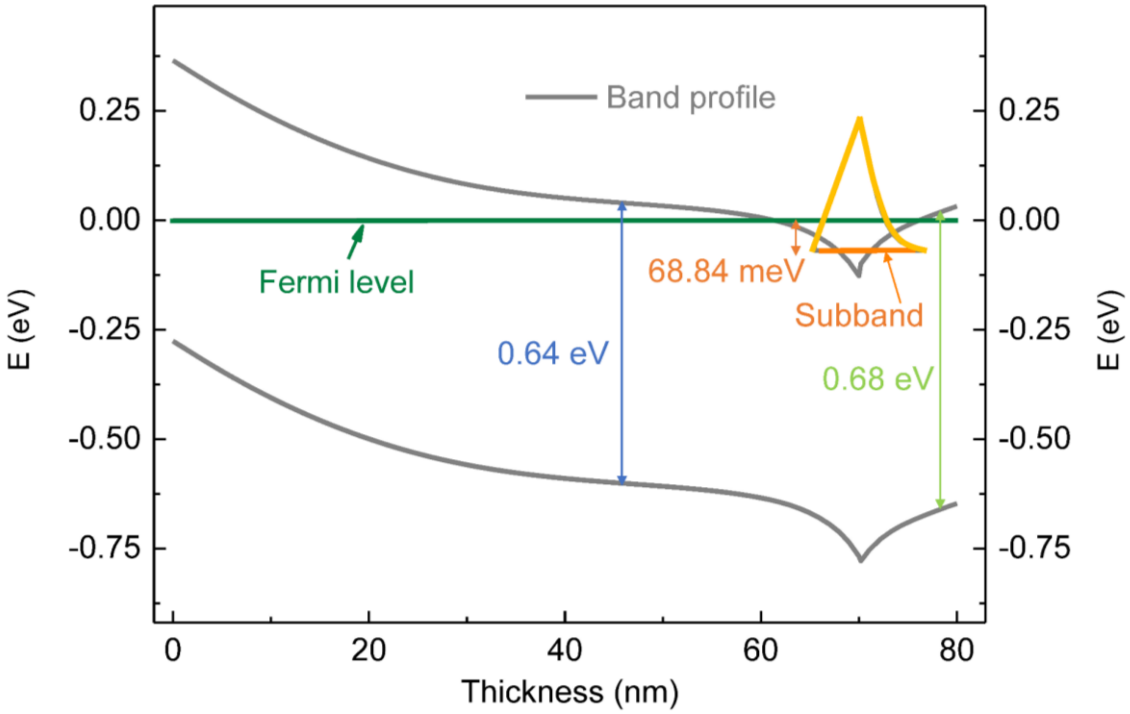

Figure S8. Band diagram of InGaN/InN at the interface. Only one subband (orange line) is occupied below the Fermi level, and the gap between the Fermi level and the subband is 68.84 meV.

To more explicitly reveal the origin of peak β, we simulated the energy band structure and subband occupation of the 2DEG in the InGaN/InN heterostructure. We carried out a simulation based on the Schrödinger and Poisson equations using an APSYS program developed by Crosslight Software, Inc. using advanced physical models. The band diagram shown in Figure. S8 indicates that only one subband was occupied below the Fermi energy level, and that the gap between the Fermi energy level and the subband was 68.84 meV. The bandgap of InN was set to 0.64 eV,[3] and the bandgap of $In_{0.96}Ga_{0.04}N$ was 0.68 eV, with the bowing parameters being 1.9 eV.[4]

References

[1] L. Hsu, R. Jones, S. Li, K. Yu, W. Walukiewicz, *J. Appl. Phys.* **2007**, *102*, 073705.

[2] Y. Zhao, H. Liu, C. Zhang, H. Wang, J. Wang, Z. Lin, Y. Xing, H. Lu, J. Liu, Y. Wang, *Physical Review X*. **2015**, *5*, 031037.

[3] J. Wu, W. Walukiewicz, W. Shan, K. Yu, J. Ager Iii, S. Li, E. Haller, H. Lu, W. J. Schaff, *J. Appl.*

Phys. **2003**, *94*, 4457.

[4] S. Liu, X. Wang, G. Chen, Y. Zhang, L. Feng, C. Huang, F. Xu, N. Tang, L. Sang, M. Sumiya, *J. Appl. Phys.* **2011**, *110*, 113514.
